# Lactate modulates the function of myeloid-derived suppressor cells via Ten-Eleven-Translocation-2-mediated demethylation of glucocorticoid-inducible kinase 1 in lung cancer model

**DOI:** 10.3389/fcell.2025.1565993

**Published:** 2025-08-22

**Authors:** Ying Chu, Hua Shen, Qiu Li, Bo Shen, Yan Zhang, Deqiang Wang, Wei Zhu, Shengjun Wang, Jie Ma

**Affiliations:** ^1^ Department of Oncology, Institute of Digestive Diseases, The Affiliated Hospital of Jiangsu University, Zhenjiang, Jiangsu, China; ^2^ Department of Immunology, Jiangsu Key Laboratory of Laboratory Medicine, Jiangsu University, Zhenjiang, Jiangsu, China; ^3^ Department of Laboratory Medicine, School of Medicine, Jiangsu University, Zhenjiang, Jiangsu, China; ^4^ The Affiliated Cancer Hospital of Nanjing Medical University, Jiangsu Cancer Hospital & Jiangsu Institute of Cancer Research, Nanjing, Jiangsu, China

**Keywords:** lactate, MDSCs, TET2, SGK1, DNA methylation

## Abstract

**Background:**

Lactate has been shown to play an important immunosuppressive role in the tumor microenvironment (TME) and promote tumor progression through a variety of different mechanisms of action. Myeloid-derived suppressor cells (MDSCs) are important cells that play an immunosuppressive role in the TME. However, the underlying mechanism by which lactate regulates MDSCs remains unclear. This study aims to explore the molecular mechanism by which lactate regulates the immunosuppressive function of MDSCs in the TME, providing new ideas and targets for anti-tumor immunotherapy targeting MDSCs.

**Methods:**

This study used the Lewis lung carcinoma cell line to establish a subcutaneous lung cancer model; MDSCs were isolated from the spleens of these mice for subsequent experiments. Protein expression was analyzed by Western blot, mRNA expression by qRT-PCR, protein-DNA interactions by ChIP-qPCR, and DNA methylation by MSP-qPCR and BSP. Exploring the regulatory mechanism of CD38 on the immunosuppressive function of MDSCs by knockdown and overexpression techniques.

**Results:**

We found that compared with spleen-derived MDSCs (SP-MDSCs) of subcutaneous lung cancer model, tumor-derived MDSCs (T-MDSCs) had stronger immunosuppressive function. Lactate could promote the immunosuppressive function of MDSCs, significantly upregulate the expression of serum and glucocorticoid-inducible kinase 1 (SGK1) in MDSCs. Further studies demonstrated that lactate could downregulate the DNA methylation level of SGK1 by regulating the Ten-Eleven-Translocation-2 (TET2) and TET2 was closely related to the immunosuppressive function of MDSCs and the progression of tumors.

**Conclusion:**

Lactate can upregulate the expression of SGK1 through demethylation mediated by TET2, enhancing the immunosuppressive function of MDSCs to promote tumor progression. It provides the effective therapeutic targets for anti-tumor therapy.

## 1 Introduction

Myeloid-derived suppressor cells (MDSCs) were first reported in the early 1970s (Talmadge et al., 2013), and were defined as bone marrow cells with immunosuppressive function (Bronte et al., 1999). MDSCs can be classified into two groups, CD11b^+^ Ly6G^−^ Ly6C^high^ monocytic MDSCs (Mo-MDSCs) and CD11b^+^ Ly6G^+^ Ly6C^low^ polymorphonuclear MDSCs (PMN-MDSCs), based on their nuclear morphology and surface markers (Bronte et al., 2016). The most important characteristic of MDSCs is their immunosuppressive properties (Joshi and Sharabi, 2022; Özbay Kurt et al., 2024; Mo et al., 2023). In the context of cancer, MDSCs are usually recruited into the tumor microenvironment to participate in tumor immune escape (Li et al., 2021). MDSCs are also involved in non-immune aspects of tumor biology, including tumor angiogenesis and metastasis (Yang et al., 2024). A large number of studies have shown a correlation between the frequency of circulating tumor-infiltrating MDSCs and tumor staging, progression and treatment resistance. (Dys et al., 2020). Hence, understanding MDSCs biology represents an important step toward enhancing anticancer immunity. In our preliminary experiments, we found that MDSCs derived from tumor tissues had a stronger immunosuppressive function than MDSCs derived from the spleen of a subcutaneous lung cancer model (Shen et al., 2019). However, the specific regulatory mechanisms involved in this process remain unclear.

Tumor cells accelerate their growth and proliferation through aerobic glycolysis, which was first described by Otto Warburg and is accompanied by the production of large amounts of lactate, leading to the formation of a low-pH state in the tumor microenvironment (TME) (Warburg, 1956). Lactate is an important molecule involved in metabolic processes and is closely related to cancer progression. Recent studies have indicated that lactate is not only a metabolic product of glycolysis, but also an energy substrate, signal molecule, and important immunosuppressive factor in tumor (Ippolito et al., 2019). In the TME, lactate can promote angiogenesis (Tao et al., 2023; Rastogi et al., 2023; Zhang et al., 2022), tumor immune escape (Zhou et al., 2024), invasion (Li et al., 2019; Zhang et al., 2021), metastasis (Paul et al., 2022)and led to poor prognosis (García-Cañaveras et al., 2019; Gharipour et al., 2020; Zong et al., 2024). It has become increasingly recognized that lactate is involved in establishing an immunosuppressive microenvironment. Lactate secreted by tumor cells can promote tumor development by inducing M2-like polarization and Arginase-1(Arg-1) expression in tumor-associated macrophages (TAMs) (Shan et al., 2020; Zhang and Li, 2020; Yan et al., 2024). Meanwhile, lactate can inhibit the maturation of dendritic cells (DCs) (Gottfried et al., 2006), induce the apoptosis of natural killer (NK) cells, and promote the immune escape of tumor cells (Kumar et al., 2019; Harmon et al., 2019). By inhibiting mTOR signaling, lactate inhibits the production of interferon-γ (IFN-γ) and interleukin-4 (IL-4) by anti-tumor natural killer T (NKT) cells in the TME (Wang et al., 2020). Increased lactate secretion can also promote the development of MDSCs and participate in the regulation of innate and adaptive immune responses (Morrot et al., 2018). Yang et al. suggested that increased lactate secretion is responsible for the enhanced immunosuppressive phenotype of MDSCs after radiotherapy (Yang et al., 2020). Lactate can aggravate immunosuppression by increasing the frequency of MDSCs, further demonstrating that lactate contributes to the establishment of an immunosuppressive microenvironment for developing tumors (Hayes et al., 2021). However, the role of lactate in the regulation of MDSCs cannot be ignored. Thus, further studies are urgently needed to identify the effects of lactate on MDSCs function.

Serum/glucocorticoid-regulated kinase 1(SGK1), a new AGC kinase family member discovered by Webster et al. (1993) in belongs to the serine/threonine protein kinase family and has high homology and kinase functions similar to those of AKT (Leroux et al., 2018). SGK1 participates in various physiological and pathological processes and its biological regulation has received widespread attention. Current research on SGK1 shows that ion channels (such as ENaC and KCNE1/KCNQ1), carriers (such as NCC and NHE3), Na (^+^)/K (^+^)-ATPase (such as GSK3), and transcriptional regulators (FOXO3a, NF-*k*B, β-catenin, and p27) are regulated by SGK1 (Lang et al., 2006). SGK1 is involved in the regulation of cell growth, proliferation, survival, migration, and tolerance to radiotherapy, chemotherapy, and other pathological processes (Talarico et al., 2016). The expression of SGK1 in cells is regulated by epigenetic factors, particularly DNA methylation. DNA methylation is an epigenetic modification with well-defined characteristics that is catalyzed by three enzymes from the DNA methyltransferase (DNMTs) family, including DNMT1, DNMT3A, and DNMT3B. DNA methylation in eukaryotes usually occurs in the cytosine of CpG dinucleotides, with S-adenosine methionine as the methyl donor and the fifth carbon atom of cytosine as the receptor for the methyl to form 5 mC. Active DNA demethylation is mediated by the Ten-Eleven-Translocation (TETs) enzymes including TET1, TET2, and TET3. Methylated 5 mC is repeatedly oxidized to 5-hydroxymethylcytosine (5hmC), 5-formylcytosine (5 fC), and 5-carboxycytosine (5caC) by TETs, and the existing methylated markers are removed by the base excision repair (BER) pathway.

In this study, we found that tumor-derived MDSCs (T-MDSCs) had a stronger immunosuppressive function than spleen-derived MDSCs (SP-MDSCs) in a subcutaneous lung cancer model. After treatment with lactate *in vitro*, MDSCs function was markedly upregulated, and further transcriptome sequencing results revealed that SGK1 was necessary for MDSCs to maintain their function. Mechanistically, we found that lactate downregulates the DNA methylation level of SGK1 via TET2. These findings suggest a novel mechanism by which lactate enhances the immunosuppressive function of MDSCs through TET2-mediated SGK1 demethylation, which may be a promising approach for tumor therapy.

## 2 Materials and methods

### 2.1 Cell and animal model

Lewis lung carcinoma cells (LCC) were preserved in our laboratory, 6–8 weeks old C57BL/6 mice were purchased from the Experimental Animal Center of Jiangsu University. The cells were cultured in DMEM (Biological Industries, Israel) supplemented with 10% fetal bovine serum at 37 °C with 5% CO_2_. A total (3 × 10^6^) LCC were inoculated under the skin of the abdominal wall of the mice to construct a mouse subcutaneous lung cancer model.

### 2.2 Purification of MDSCs and CD4^+^ T cells

The spleen from tumor-bearing mice was minced into small pieces and the suspension was obtained by grinding the spleen in a sieve with a grinder. Red blood cell lysis buffer (ACK) was added to the suspension to remove red blood cells (RBCs). Tumor masses from tumor-bearing mice were minced into pieces, digested in RPMI-1640 (Biological Industries, Israel) containing 0.5 mg/mL collagenase, hyaluronidase and DNase I (Sigma-Aldrich, St. Louis, MO) at 37 °C for 2 h. To obtain high-purity CD11b^+^ Gr1^+^ cells, Mouse MDSCs isolation kit (Miltenyi Biotec, Auburn, CA) was used according to the manufacturer’s instructions. Murine CD4^+^ T cells were isolated from the spleens of wild-type C57BL/6 mice using a Mouse CD4^+^ T cell Isolation Kit (Miltenyi Biotec, Auburn, CA, United States of America). The purity of MDSCs and CD4^+^ T cells was determined by flow cytometry (FCM).

### 2.3 RNA sequencing and differential gene expression analysis

The spleen-derived MDSCs from wild-type mice were sorted using immunomagnetic beads *in vitro* and divided into three groups according to different treatment factors: control group (group C), pH 6.7 group (Concentrated hydrochloric acid was added to the cell culture medium to adjust the pH to 6.7, group 6_7), and lactate treatment group (group L). Group 6_7 was used to exclude the effects of the acidic environment. Total RNA was extracted from cells for whole-genome transcription detection (Commissioned by Shengong Bioengineering Shanghai Co., LTD., Contract No. MRNA190213NJ). Bioinformatics analysis was performed on the sequencing results, including analysis of differentially expressed genes and KEGG database analysis.

### 2.4 Western blot analysis

Cell and tissue lysates were separated by SDS-PAGE, transferred to nitrocellulose or polyvinylidene difluoride (PVDF) membranes (Bio-Rad, Hercules, CA, United States of America), and blotted with primary antibodies overnight at 4 °C, followed by incubation with secondary HRP-conjugated antibodies. Immunoblots were visualized using the LAS4000 Chemiluminescence gel imaging and analysis system (Champion Chemical, Whittier, CA, United States of America). The following antibodies were used: monoclonal antibodies specific for SGK1 and TET2 were purchased from Abcam (Cambridge, United Kingdom), and monoclonal antibodies specific for ββ-actin were obtained from Cell Signaling Technology (Beverly, MA, United States of America).

### 2.5 Reverse transcription-polymerase chain reaction (RT-PCR)

Total RNA from cultured or sorted cells was purified using TRIzol reagent (Thermo Fisher Scientific, Waltham, MA, United States of America) and reverse-transcribed into cDNA using the SuperScript VILO cDNA Synthesis Kit (Thermo Fisher Scientific). The KAPA SYBR FAST qPCR Kit (Kapa Biosystems, Wilmington, MA, United States of America) and the corresponding primer sets were used for qRT-PCR analysis using the Bio-Rad CFX96 Real-time System (Bio-Rad, Hercules, CA, United States of America). The relative expression was normalized to that of β-actin and calculated using the 2^−ΔΔCT^ method.

SGK1-F:5′- GGCTAGGCACAAGGCAGAAGAAG-3′

SGK1-R:5′- GCGGTCTGGAATGAGAAGTGAAGG-3′

HK2-F:5′- ATGCGGCTCTCTGATGAAAT-3′

HK2-R:5′- GACAAAGGTTGGCAGCATCT-3′

PKM2-F:5′-GGGAGCCACTCTCAAAATCA-3′

PKM2-R:5′-ACCTTTTCTGCTTCACCTGGA-3′

PFK-F:5′- GCCTGAAAGCTGCCTGTAAC-3′

PFK-R:5′- AGAAGTCCGCTCCACTCCTT-3′

GLUT1-F:5′-AAACATGGAACCACCGCTAC-3′

GLUT1-R:5′-AACAAAGAGGCCGACAGAGA-3′

LDHA-F:5′-GAGGTTCACAAGCAGGTGGT-3′

LDHA-R:5′-ACCCGCCTAAGGTTCTTCAT-3′

HIF1-α-F:5′- AGCCCTAGATGGCTTTGTGA-3′

HIF1-α-R:5′- TATCGAGGCTGTGTCGACTG-3′

MCT1-F:5′-AAACATGGAACCACCGCTAC-3′

MCT1-R:5′-AACAAAGAGGCCGACAGAGA-3′

MCT4-F:5′-GAGGTTCACAAGCAGGTGGT-3′

MCT4-R:5′-ACCCGCCTAAGGTTCTTCAT-3.

DNMT3A-F:5′- GATGATCGAAAGGAAGGAGAGG-3′

DNMT3A-R:5′- TTCTCCAAGTCTCCATTGGGTA-3′

DNMT3B-F:5′-GCTCTTCTTCGAGTTTTACCAC -3′

DNMT3B-R:5′-ATCATTCTTTGAAGCCATCACG -3′

TET1-F:5′- CTAGTCCATCTGAGCAGCTAA-3′

TET1-R:5′- GGAAGCCAGTTTCTGAGAATTG -3′

TET2-F:5′- CTGCTGTTTGGGTCTGAAGGAAGG-3′

TET2-R:5′- GTTCTGCTGGTCTCTGTGGGAATG-3′

TET3-F:5′- GAACCTGACGGCACGAGCAAG-3′

TET3-R:5′- TGTGGACTGTGGAGACGGATGG-3′

β-actin-F:5′- GTGCTATGTTGCTGCTCTAGACTTCG-3′

β-actin-R:5′- ATGCCACAGGATTCCATACC-3′

### 2.6 Detection of immunosuppressive function of MDSCs

MDSCs were co-cultured with CD4^+^ T cells derived from the spleen, which were labeled with the fluorescent dye CFSE (5 μM, Invitrogen) in 96-well round-bottomed plates in the presence of anti-CD3 mAb and anti-CD28 mAb (BioLegend, San Diego, CA, United States of America) for 72 h. The proliferative ability of CD4^+^ T cells was detected using FCM to estimate the suppressive activity of MDSCs. The co-culture cell ratio (MDSCs:CD4^+^ T cells) is 1:1.

### 2.7 Measurement of Arg-1 activity

MDSCs were lysed with an appropriate amount of RIPA buffer for 30 min and the lysate supernatant was collected after centrifugation. The QuantiChrom Arginase Assay Kit (BioAssay Systems, Hayward, CA, United States of America) was used to determine Arg-1 activity in the lysate supernatants.

### 2.8 Measurement of NO content

MDSCs were cultured in 24-well plates with RPMI 1640 medium containing 10% fetal bovine serum and transfected with siRNA for 24 h. Then, the cells were collected, centrifuged, and the supernatant was retained. The NO content in the supernatant was evaluated using the Griess Reagent System kit (Promega, Madison, WI) according to the manufacturer’s instructions.

### 2.9 Flow cytometry

Single-cell suspensions were stained with monoclonal antibodies specific for CD11b and Gr-1 (BioLegend). For the ROS production assay, DCFDA, a ROS sensitive fluorescent dye, was used in MDSCs for 30 min at 37 °C as the ROS detection probe, and the fluorescence intensity was quantified using flow cytometry. Apoptosis was assayed using the Annexin V-FITC Apoptosis Detection Kit (Invitrogen, Carlsbad, CA, United States of America) according to the manufacturer’s instructions. MDSCs were collected following incubation with lactate for 24 h, and the expression levels of CD86, MHC II, CD80, CD40, and F4/80 (eBioscience, San Diego, CA) were assessed by FCM.

### 2.10 Assays for lactate release

The concentrations of glucose and lactate in the culture medium were measured using a Glucose Test Kit (Invitrogen, Carlsbad, CA, United States of America) and lactic acid Assay Kit (Jiancheng Bioengineering, Nanjing, China) individually after collecting the supernatant of MDSCs. The supernatant was added to the reagent and the mixture was incubated for 10 min at 37 °C. Standard reagent and reagent blank were provided by the manufacturer and strictly followed the *manufacturer’s instructions*. Absorbance was quantified spectrophotometrically at 340 nm and 530 nm. Glucose uptake and lactate release were calculated using formulas provided by the manufacturer.

### 2.11 Trypan blue dying

Planted 2 × 10^6^ MDSCs in a 24-well plate, and different treatments were added to the culture system for 24 h. Cells were resuspended in PBS buffer, and 5 μL of 4% Trypan Blue staining solution was added and incubated for 3–5 min. After incubation time is reached, the cell suspension was added to a beef abalone counting plate and the cells were counted under a microscope.

### 2.12 CpG island prediction

CpG island prediction and methylation detection in the promoter region of SGK1 gene. Online CpG island prediction software (http://www.urogene.org/methprimer) was used to detect the upstream and downstream 1 kb promoter regions of the mouse SGK1 gene (https://www.ncbi.nlm.nih.gov/nuccore/NC_000076.6?from=21882184&to= 21,999,903 &report = GenBank) and found a CpG island with a length of 213 bp.

### 2.13 The methylation level of SGK1 was detected by methylation specific PCR (MSP)

Genomic DNA was extracted using the Trelief ®Animal Genomic DNA Kit (Tsingke Biotechnology, Beijing, China), according to the manufacturer’s protocol. Sodium bisulfite conversion of genomic DNA was performed using a BisulFlash™ DNA Modification Kit (Epigentek, United States of America). Bisulfite-converted genomic DNA was amplified using EpiTect Master Mix for methylation-specific PCR (MSP, Qiagen) with methylation-specific or unmethylation-specific primers. The PCR products were analyzed by electrophoresis.

Left M primer: GTCGTTCGTTTTTCGTTTAGTTC.

Right M primer: CTACGACAACGACTACAATAACTCG.

Left U primer: TTGTTTGTTTTTTGTTTAGTTTGG.

Right U primer: ACAACAACAACTACAATAACTCACC.

### 2.14 The methylation level of SGK1 was detected by bisulfite sequencing PCR (BSP)

Genomic DNA extraction and bisulfite treatment were performed as described for the MSP method. PCR was performed to amplify the CpG island fragment of the SGK1 promoter from the bisulfite-converted genomic DNA using BSP primers. The PCR products were then cloned and sequenced. Shengong Bioengineering Shanghai Co., Ltd. was used to analyze methylation levels.

BSP-F: AAAATAGTTTTTTTTTAGTAGTTTTTAGTAG.

BSP-R: ATTTTTCACTCRCTCATCCTACTAC.

### 2.15 Transfection of small interfering RNA

The siRNA working solution and transfection reagent working solution according to a 50 μL system per well (2 μL siRNA + Lipofectamine 2000 + 48 μL serum-free 1,640 medium), mixed, and incubated at room temperature for 10 min. The transfection reagent working solution was added to the siRNA working solution, mixed, and incubated at room temperature for 20 min. The cells were added to a 24-well plate and placed in an incubator for 4–6 h. Observed the cell status after 4–6 h, the cells were cultured in RPMI 1640 medium containing 20% FBS, 500 μL was added to each well for rehydration and culturing was continued according to the requirements of the experiment.

### 2.16 Chromatin immunoprecipitation PCR analysis

Chromatin immunoprecipitation (ChIP) assay was performed using the Simple ChIP Enzymatic Chromatin IP Kit (Magnetic Beads, Cell Signaling Technology) according to the manufacturer’s instructions. The disposed chromatin was subjected to immunoprecipitation with a normal IgG antibody (rabbit; Cell Signaling Technology, 2729S; RRID: AB_1031062, Beverly, MA) as a negative control (NC), histone H3 antibody (rabbit; Cell Signaling Technology, 4620S; RRID: AB_1904005, Beverly, MA, United States of America) as a positive control, and TET2 antibody (mouse; 21207-1-AP, Proteintech, United States of America). For ChIP-PCR, DNA samples and standards were analyzed using SimpleChIP® Universal qPCR Master Mix (#88989, Cell Signaling Technology, Beverly, MA, United States of America) on the Bio-Rad CFX96 Real-time System (Bio-Rad, Hercules, CA, United States of America).

Take the PIC and 10×ChIP solution from the ChIP detection kit at −80 °C refrigerator and preheat to prepare 3 mL of low-salt washing solution (300 μL of 10×ChIP buffer + 2.7 mL of double-distilled water). Prepare 1 mL of high-salt washing solution (100 μL of 10×ChIP buffer + 0.9 mL of double-distilled water + 70 μL of 5 mmol/L NaCl). After the above solutions are prepared, store them at room temperature.

For each immunoprecipitation, add 400 μL of ChIP buffer. Positive controls and negative controls were set up respectively. Add 100 μL of cross-linked chromatin preparation. Take 10 μL of chromatin preparation into a new 1.5 mL centrifuge tube and use it as an additional 2% input sample. Add TET2 ChIP antibody. 10 μL of H3 positive control antibody was added to the positive group. In the negative group, 1 μL (1 µg) to 2 μL (2 µg) of rabbit IgG negative control antibodies were added. Incubate overnight in a 4 °C refrigerator. Add 30 μL of Protein G Magnetic Beads to each tube and incubate by rotation in a 4 °C refrigerator for 2 h. Transfer the reactants to a test tube and place it in a small magnetic separation rack to ensure that all the magnetic beads are adsorbed and aggregated. Carefully discard the clear supernatant after several minutes. Add 1 mL of low-salt washing solution to the test tube and repeat twice. Add 1 mL of high-salt washing solution to the test tube to wash the precipitate and incubate for 5 min. Place the test tube in a small magnetic separation rack to ensure that all the magnetic beads are adsorbed and aggregated. Carefully discard the clear supernatant after several minutes.

### 2.17 Statistical analysis

The experimental data are expressed as the mean ± SEM. Student’s t-test and ANOVA were used to determine the statistical differences. The differences at p < 0.05 were considered statistically significant.

## 3 Results

### 3.1 Tumor-derived MDSCs (T-MDSCs) have stronger immunosuppressive function than spleen-derived MDSCs (SP-MDSCs) of mouse subcutaneous lung cancer model

**FIGURE 1:**
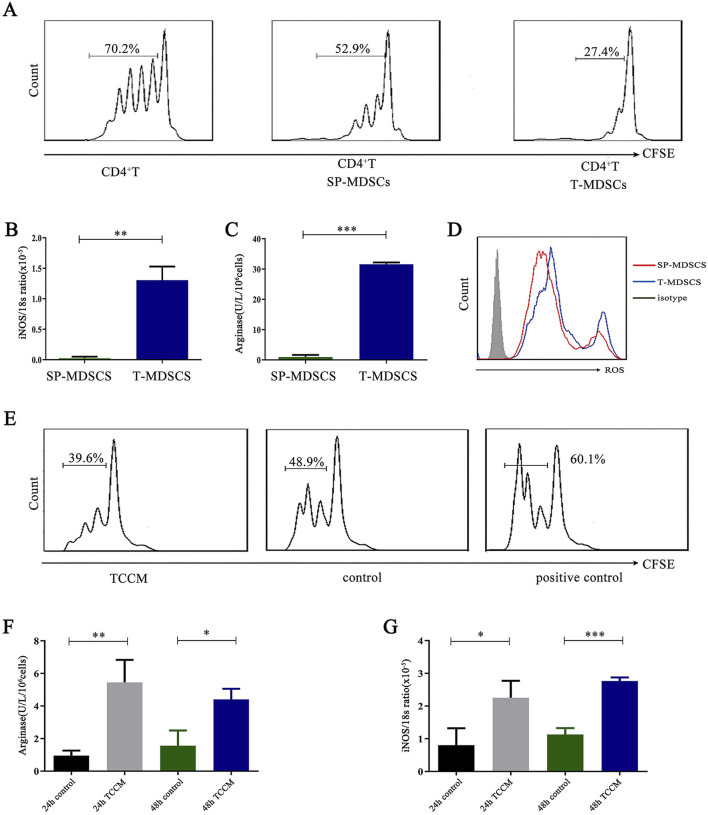
T-MDSCs have stronger immunosuppressive function than SP-MDSCs of mouse subcutaneous lung cancer model. **(A)** Approximately 1 × 10^6^ LLC cells were injected under the backs of mice by s. c. to establish mouse subcutaneous lung cancer model (n = 3 per group). SP-MDSCs from tumor-bearing mice were isolated by immunomagnetic beads from spleen and T-MDSCs were isolated by FCM from tumor tissue of mouse subcutaneous lung cancer model. CFSE-labeled CD4^+^ T cells were co-cultured with SP-MDSCs and T-MDSCs in the presence of CD3 and CD28 stimulation respectively, the co-culture cell ratio (MDSCs:CD4^+^ T cells) is 1:1 (n = 3 per group). After 72 h, the proliferation of CD4^+^ T cells was detected by FCM. **(B)** The expression level of iNOS was detected by a Griess reagent system kit (n = 3 per group). **(C)** The activity of Arg-1 was detected by a QuantiChrom arginase assay kit (n = 3 per group). **(D)** The expression level of ROS was detected by FCM (n = 3 per group). **(E)** The proliferation of CD4^+^ T cells was detected by FCM after MDSCs were treated by TCCM. After 72 h, the proliferation of CD4^+^ T cells was detected by FCM (n = 3 per group). **(F,G)** The activity of Arg-1 was measured by a QuantiChrom arginase assay kit, and the concentration of NO was determined by a Griess reagent system kit after MDSCs were treated by TCCM for 24 h and 48 h respectively (n = 3 per group). The results are presented as means ± SEM, *p < 0.05, **p < 0.01, ***p < 0.001. T-MDSCs: MDSCs derived from the tumor tissue of mouse subcutaneous lung cancer model. SP-MDSCs: MDSCs derived from the spleens of mouse subcutaneous lung cancer model. TCCM: Tumor cell conditioned medium.

In the middle and late stages of the tumor, a large number of MDSCs accumulate in the tumor tissue (Ma et al., 2018). We were curious about the differences between MDSCs from different tissues, especially from the spleen and tumors. Therefore, we purified SP-MDSCs and T-MDSCs in a mouse lung cancer xenograft model and co-cultured them with CD4^+^ T cells (1:1) derived from the spleen of wild-type mice. The results showed that, compared to SP-MDSCs, T-MDSCs had a stronger immunosuppressive effect on the proliferation of CD4^+^ T cells (Figure 1A). To further confirm the immunosuppressive function of MDSCs, we measured the expression levels and activities of immunosuppressive effector molecules include inducible nitric oxide synthase (iNOS) (Figure 1B), and Arg-1 (Figure 1C), and reactive oxygen species (ROS) (Figure 1D). These results show that the expression level or activity of all these factors in T-MDSCs was higher than that in SP-MDSCs. Furthermore, to clarify whether these differences are due to the different tissue microenvironments, we used Lewis lung cancer cell (LCC) tumor cell-conditioned medium (TCCM) to simulate the tumor microenvironment and treated SP-MDSCs *in vitro*. The results showed that, after treatment with TCCM, the immunosuppressive function of MDSCs was significantly upregulated (Figure 1E), and iNOS and Arg-1 expression was greatly enhanced (Figures 1F,G).

There is a complex correlation between cell metabolic state and biological function. It is worth mentioning here that the differences between T-MDSCs and SP-MDSCs are not only the immunosuppressive function but also the level of glycolysis. The results showed that compared with SP-MDSCs, the expression level of hexokinase 2 (HK2), phosphofructokinase (PFK) and lactate dehydrogenase A (LDHA) were significantly upregulated in T-MDSCs (Supplementary Figure S1A). Concurrently, glycolytic pathway-related proteins, including glucose transporter 1 (GLUT1) and hypoxia-inducible factor-1 (HIF-1), were more abundant in T-MDSCs (Supplementary Figure S1B). Similarly, we used TCCM to simulate the TME and treat SP-MDSCs *in vitro*. The results showed that MDSCs absorbed more glucose and released more lactate after treatment with TCCM (Supplementary Figure S1C, S1D). MDSCs expressed higher mRNA levels of glycolytic pathway-related molecules, including LDHA, GLUT1, and HIF-1 (Supplementary Figure S1E).

In conclusion, T-MDSCs have a stronger immunosuppressive function than SP-MDSCs because of the TME. Then, we tend to identify what kind of the factor in the TME would regulate the immunosuppressive function of MDSCs.

### 3.2 Lactate enhances the immunosuppressive function of MDSCs

**FIGURE 2:**
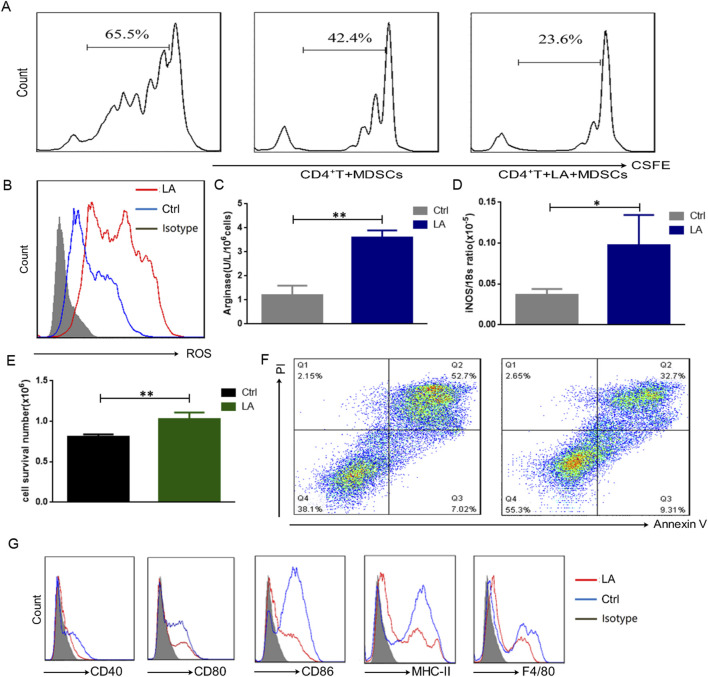
Lactate enhances the immunosuppressive function of MDSCs. **(A)** The immunosuppressive functions of MDSCs from wild-type mice were tested by the proliferating of CFSE-labeled CD4^+^ T cells, which was measured by FCM after 72 h (n = 3 per group). **(B)** The expression level of ROS was detected by FCM after treatment by lactate (10 μM) for 24 h (n = 3 per group). **(C)** The activity of Arg-1 was measured by a QuantiChrom arginase assay kit after treatment by lactate (10 μM) for 24 h (n = 3 per group). **(D)** The concentration of NO was determined by a Griess reagent system kit after treatment by lactate (10 μM) for 24 h (n = 3 per group). **(E)** The survival cells were counted by the trypan blue analysis (n = 3 per group). **(F)** Apoptosis of MDSCs was detected by FCM after treatment by lactate (10 μM) for 24 h (n = 3 per group). **(G)** The surface markers of MDSCs were detected by FCM (n = 3 per group). The results are presented as means ± SEM, *p < 0.05, **p < 0.01. LA: lactate, LA + MDSCs: SP-MDSCs was treated by lactate for 24 h.

The TME has very complex components, including a variety of metabolites, the most noteworthy of which is lactate, which is mainly secreted by tumor cells and accumulates in large quantities in the TME (Wang et al., 2023). To further reveal the mechanism that mediates the immunosuppressive function of MDSCs, we treated SP-MDSCs from wild-type mice with lactate (10 μM) for 24 h and then co-cultured them with CD4^+^ T cells for 72 h. Results showed that the proliferation of CD4^+^ T cells was obviously decreased, which meant that the immunosuppressive function of MDSCs was significantly increased (Figure 2A). Consistently, the secretion of immunosuppressive effector molecules such as ROS, Arg-1, and iNOS was significantly increased (Figures 2B–D).

Furthermore, the expression of key molecules involved in the glycolytic pathway, including HK, PFK, and LDHA, was significantly increased after treatment with lactate (Supplementary Figure S2A). Concurrently, glycolytic pathway-related proteins, including GLUT1 and HIF-1, were upregulated (Supplementary Figure S2B). MDSCs could take up more glucose and release more lactate (Supplementary Figure S2C, S2D), indicating that lactate could also upregulate glycolysis in MDSCs.

We were curious whether the enhancement of glycolysis contributed to the increased immunosuppressive function of MDSCs. We used 2-deoxy-D-glucose (2-DG) to block the glycolysis pathway in MDSCs before functional detection. The results showed that the expression of key molecules in the glycolytic pathway, including HK, PFK, and LDHA, was significantly decreased after 2-DG treatment (Supplementary Figure S3A). Concurrently, glycolytic pathway-related proteins, including GLUT1 and HIF-1, were also decreased (Supplementary Figure S3B), accompanied by reduced glucose uptake and lactate production (Extended Data Figures 3C,D). Consistent with these expectations, the activity of Arg-1 (Supplementary Figure S3E), and iNOS expression (Supplementary Figure S3F) were significantly reduced after the addition of 2-DG, suggesting that the immunosuppressive function of MDSCs was inhibited.

**FIGURE 3:**
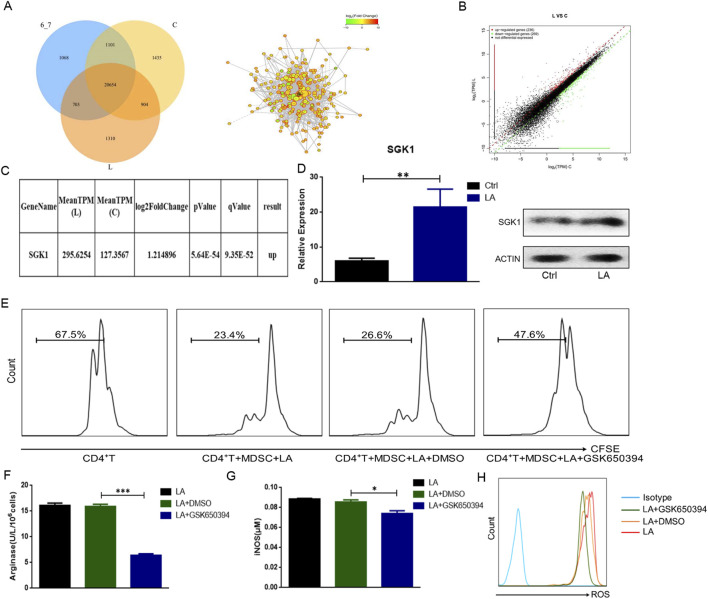
Lactate enhances the immunosuppressive function of MDSCs by regulating SGK1. **(A)** Differentially expressed genes were screened by transcriptome sequencing. The protein-protein interaction network analyzed in String data base. Venn diagram showed the number of co-expressed genes in the three groups. **(B)** The difference of gene expression between group L and group C was shown in scatter plot. **(C)** Whole genome transcriptional sequencing showed that compared with group C the expression of SGK1 was significantly increased after treatment of lactate. **(D)** The expression level of SGK1 was verified by qRT-PCR and Western blot respectively. **(E)** The proliferation of CD4^+^ T cells was detected by FCM after adding GSK690394 (10 μM) to the culture system for 24 h (n = 3 per group). **(F)** The activity of Arg-1 was measured by a QuantiChrom arginase assay kit (n = 3 per group). **(G)** The concentration of NO was determined by a Griess reagent system kit (n = 3 per group). **(H)** The expression level of ROS was detected by FCM (n = 3 per group). The results are presented as means ± SEM, *p < 0.05, **p < 0.01, ***p < 0.001. Two-tailed Student’s t-test was used for two-group comparisons, and one-way ANOVA with an LSD post hoc test was used for multi-group comparisons. LA: lactate, GSK690394: an inhibitor of SGK1. Group **(C)** control group, group L: lactate treatment group.

In addition, Trypan blue dye analysis showed that the survival rate of MDSCs was significantly increased (Figure 2E), and apoptosis test results revealed that the apoptosis rate of MDSCs decreased significantly in the presence of lactate (Figure 2F). Interestingly, after treatment with lactate for 24 h, the expression of surface markers of SP-MDSCs, such as CD40, CD80, CD86, MHC-II, and F4/80 decreased, indicating that lactate could maintain MDSCs in an immature state and promote their survival (Figure 2G).

Taken together, these data raise the possibility that the TME contributes to the function, glycolysis, maturity, and survival of MDSCs, and that this contribution is associated with a large amount of lactate in the TME.

### 3.3 Lactate regulates the function of MDSCs by up-regulating SGK1

To clarify which genes are involved in regulating the function of MDSCs by lactate, SP-MDSCs from tumor-bearing mice were sorted using immunomagnetic beads *in vitro* and divided into three groups according to different treatment factors: control group (group C), pH 6.7 group (Concentrated hydrochloric acid was added to the cell culture medium to adjust the pH to 6.7) (group 6_7), and lactate treatment group (group L). The results of whole-genome transcription sequencing showed that the number of genes co-expressed in these three groups was 20,654, co-expressed in group C and group L was 21,558. The number of genes specifically expressed in group L was 1,310 (Figure 3A). Compared to group C, the expression of 505 genes in group L was significantly changed, among which 236 genes were upregulated and 269 genes were downregulated (Figure 3B).

We analyzed the results and found that the expression of SGK1 was significantly higher in group L than in group C (Figure 3C). To confirm the sequencing results, SP-MDSCs were isolated and treated with lactate, and SGK1 expression was verified using qRT-PCR and Western blotting (Figure 3D). These results were consistent with the transcriptional sequencing results, which showed with group C, both the mRNA and protein expression levels of SGK1 significantly increased after treatment with lactate. To further explore the mechanism underlying the effect of lactate on MDSC function through SGK1, we added the SGK1 inhibitor GSK650394 (10 μM) to the culture system (with lactate) for 24 h. The results of the CD4^+^ T cell proliferation test showed that compared to the control group, the immunosuppressive function of MDSCs on CD4^+^T cell proliferation was significantly downregulated after the addition of GSK650394 (Figure 3E). The activity of Arg-1 and the expression of iNOS and ROS significantly decreased (Figures 3F–H). Taken together, these *in vitro* findings suggested that SGK1 is a key gene for lactate enhancement in the immunosuppressive function of MDSCs.

### 3.4 Lactate decreases the methylation level of SGK1 in MDSCs

According to the above results, lactate enhances the immunosuppressive function of MDSCs by upregulating SGK1; however, the molecular mechanism remains unclear. DNA methylation is an epigenetic modification related to the inhibition of gene transcription. SGK1 expression is regulated by DNA methylation in a variety of cells. To investigate the potential regulation of SGK1 by DNA methylation in MDSCs, the SGK1 promoter region sequence was retrieved from GenBank (https://www.ncbi.nlm.nih.gov/nuccore/NC_ 000076.6. from=21882184&to=21999903&report= GenBank) and its methylation sites were predicted using MethPrimer (https://www.ncbi.nlm.nih.gov/nuccore/NC_ 000076.6. from=21882184 &to=21999903 &report= GenBank), and the methylation site of this region was predicted using the website (http://www.urogene.org/methprimer).

**FIGURE 4:**
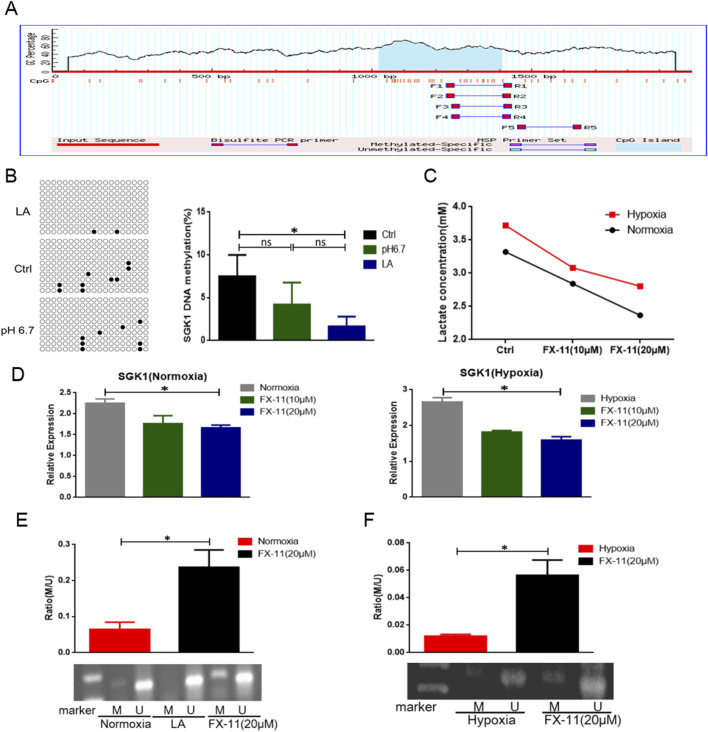
Lactate mediated demethylation of SGK1. **(A)** CpG islands of DNA methylation were found in SGK1 promoter region and the nucleotide sequence of methylated CpG islands. **(B)** DNA methylation level of SGK1 transcription initiation region was detected by BSP. **(C)** Intracellular lactate concentration was measured by lactate testing kit. **(D)** The expression level of SGK1 was tested by qRT-PCR after adding different concentration of FX-11 (10 μM, 20 μM) under both normoxia and hypoxia (n = 3 per group). **(E)** Detection of SGK1 methylation by MSP under normoxia after adding FX-11 (20 μM) (n = 3 per group). U, unmethylated; M, methylated. **(F)** Detection of SGK1 methylation by MSP under hypoxia after adding FX-11 (20 μM) (n = 3 per group). The results are presented as means ± SEM, *p < 0.05, ***p < 0.001. NS: not significant. Two-tailed Student’s t-test was used for two-group comparisons, and one-way ANOVA with an LSD post hoc test was used for multi-group comparisons. FX-11: an inhibitor of LDHA.

The prediction results showed that the CpG islands had multiple methylation sites, indicating that SGK1 could be regulated by DNA methylation (Figure 4A). Next, we performed bisulfite sequencing PCR (BSP) to analyze the methylation level of SGK1. Three groups were established: the control group (Ctrl), pH 6.7 acid treatment group (pH 6.7), and lactate treatment group (LA). SP-MDSCs were sorted using immunomagnetic beads *in vitro*, and the total DNA of each group was extracted and subjected to sodium bisulfite conversion treatment. The BSP primers were designed using the website (http://www.urogene.org/methprimer) and the target fragment was amplified. The PCR-amplified products were cloned and sequenced to analyze the degree of methylation. The results showed that compared to the pH 6.7 group, the methylation level of SGK1 in the LA group was significantly decreased (Figure 4B), suggesting that lactate could reduce the DNA methylation levels of SGK1 CpG islands.

To further confirm that lactate regulate the methylation level of SGK1, SP-MDSCs from wild-type mice were cultured under normoxic and hypoxic conditions. The LDHA inhibitor FX-11 (20 μM) was added to the culture system to inhibit lactate formation and the concentration of intracellular lactate was determined. As shown in Figure 4C, lactate production was significantly reduced by the addition of FX-11 and exhibited a concentration-dependent effect. qRT-PCR assay results showed that the mRNA level of SGK1 was significantly decreased under both normoxic and hypoxic conditions (Figure 4D). Methylation-specific PCR (MSP) assays showed that the methylation level of SGK1 was upregulated under both normoxic and hypoxic conditions after treatment with FX-11 (Figures 4E,F). Thus, lactate is involved in the regulation of SGK1 CpG island methylation levels.

### 3.5 TET2 mediates the demethylation of SGK1

**FIGURE 5:**
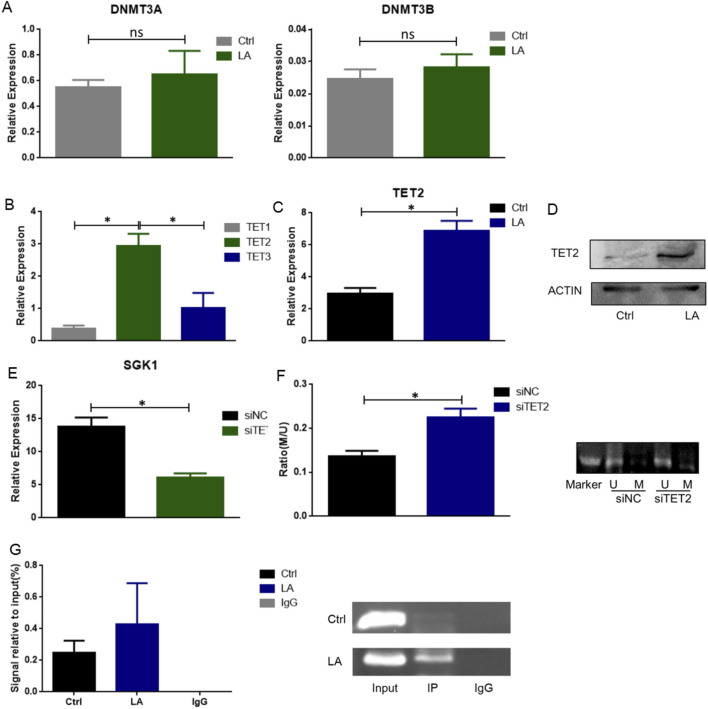
TET2 is involved in mediating SGK1 demethylation. **(A)** The expression level of DNMT3A and DNMT3B in MDSCs from wild-type mice was detected after lactate (10 μM) treatment by qRT-PCR (n = 3 per group). **(B)** The expression level of TET1, TET2 and TET3 in MDSCs from wild-type mice was detected by qRT-PCR. **(C)** The expression level of TET2 after lactate treatment was detected by qRT-PCR (n = 3 per group). **(D)** The expression level of TET2 after lactate treatment was detected by Western blot (n = 3 per group). **(E)** SiTET2 transfection efficiency was verified by qRT-PCR (n = 3 per group). **(F)** The DNA methylation level of SGK1 after siTET2 transfection was measured by MSP (n = 3 per group); U, unmethylated; M, methylated. **(G)** The binding of TET2 and SGK1 was detected by ChIP-qPCR. The results are presented as means ± SEM, *p < 0.05. NS, not significant. Two-tailed Student’s t-test was used for two-group comparisons. LA: lactate. SiNC: siRNA Control; siTET2: siRNA-TET2.

The regulation of DNA methylation is primarily mediated by methylase, DNMTs, and demethylase, TETs. To explore whether DNMTs and TETs are involved in the lactate-mediated SGK1 demethylation process, we used qRT-PCR to detect the mRNA expression of DNMTs and TETs after the treatment of SP-MDSCs from wild-type mice with lactate. The results showed that the expression levels of DNMT3A and DNMT3B did not change significantly (Figure 5A). However, compared with TET1 and TET3, TET2 was not only highly expressed in SP-MDSCs, but also increased significantly after treatment with lactate (Figures 5B,C).

To further confirm whether TET2 plays an important role in lactate-regulated demethylation of SGK1, SP-MDSCs were sorted *in vitro* and siRNA-TET2 (siTET2) was added after lactate treatment. qRT-PCR assay results showed that siTET2 effectively downregulated the mRNA expression level of SGK1 in MDSCs (Figure 5D), and the MSP results showed that the level of SGK1 DNA methylation was significantly increased after siTET2 transfection (Figure 5E). Consistently, ChIP-qPCR analysis of MDSCs showed that TET2 directly binds to the transcription initiation region of SGK1 and mediates demethylation of SGK1, which is further enhanced after treatment with lactate (Figure 5F).

### 3.6 TET2-mediated demethylation of SGK1 alleviates tumor development by decreasing the function of MDSCs

**FIGURE 6:**
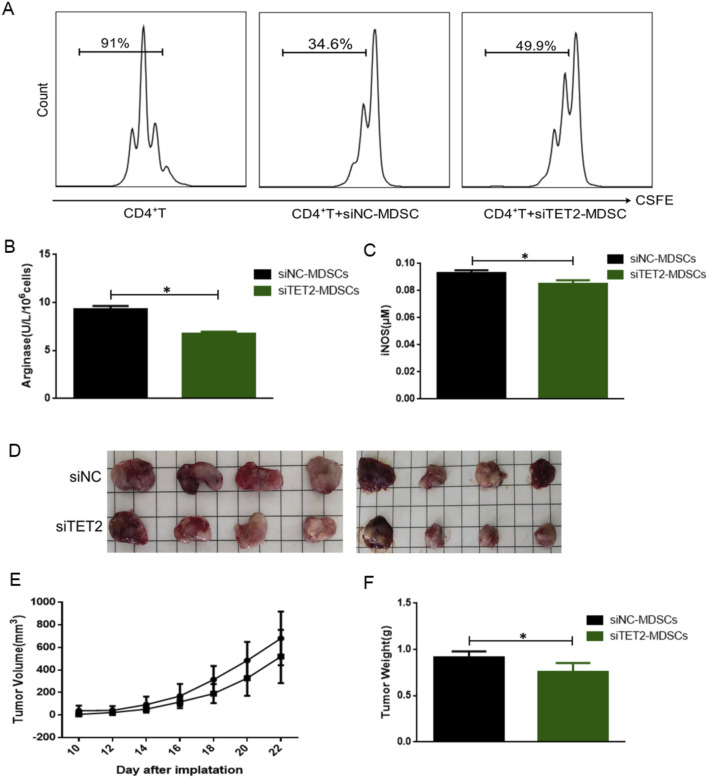
Effects of TET2-mediated SGK1 demethylation on the immunosuppressive function of MDSCs in vitro and in vivo. **(A)** The immunosuppressive function of MDSCs from wild-type mice on CD4^+^T cells after siTET2 transfection was detected by FCM (n = 3 per group). **(B)** The activity of Arg-1 was measured by a QuantiChrom arginase assay kit after siTET2 transfection (n = 3 per group). **(C)** The concentration of NO was determined by a Griess reagent system kit after siTET2 transfection (n = 3 per group). **(D)** Comparison of tumor size between siNC-MDSCs group and siTET2-MDSCs group (n = 3 per group). **(E)** The growth curve of the tumor of siNC-MDSCs group and siTET2-MDSCs group (n = 3 per group). **(F)** The weight of the tumor of siNC-MDSCs group and siTET2-MDSCs group (n = 4 per group). The results are presented as means ± SEM, *p < 0.05. Two-tailed Student’s t-test was used for two-group comparisons. SiNC-MDSCs: MDSCs transfected with siRNA Control; siTET2-MDSCs: MDSCs transfected with siRNA-TET2.

Next, we explored the therapeutic potential of targeting the demethylation SGK1 in the immunosuppressive function of MDSCs in a mouse subcutaneous lung cancer model. After treatment with siTET2, the immunosuppressive effect of MDSCs on CD4^+^ T cell proliferation was significantly downregulated (Figure 6A). Consistently, the activity of Arg-1 and expression of iNOS were significantly decreased (Figures 6B,C), indicating that TET2-mediated SGK1 demethylation was involved in the regulation of the immunosuppressive function of MDSCs *in vitro*.

To further investigate the effect of TET2-mediated SGK1 demethylation *in vivo*, we constructed a mouse subcutaneous lung cancer model and randomly divided the mice into two groups: siNC-MDSC (MDSCs transfected with siNC) and siTET2-MDSCs (MDSCs transfected with siTET2-MDSCs). MDSCs transfected with siNC/siTET2 and mouse LCC were injected subcutaneously at a ratio of 1:1, and the tumor was observed every 2 days. The long and short diameters of the tumors were measured with a Vernier caliper, tumor volume was calculated, and a growth curve was drawn. Compared with the siNC-MDSCs group, the tumor size of the siTET2-MDSCs group was reduced, and the tumor volume was decreased in the siTET2-MDSCs group (Figures 6D,E), suggesting that interfering with the expression of TET2 in MDSCs could delay tumor growth. Tumor nodules were stripped when the mice were euthanized. The weight of the tumor nodules was also greatly decreased in the siTET2-MDSCs group (Figure 6F). Collectively, our data show that compared to normal conditions, tumor cells in the TME produce a large amount of lactate through aerobic glycolysis. Lactate upregulates the expression of TET2, promotes the binding of TET2 to the CpG island of SGK1, and mediates SGK1 demethylation SGK1 demethylation leads to a significant upregulation of SGK1 expression, which participates in the regulation of immunosuppressive function and glucose metabolism in MDSCs.

## 4 Discussion

A substantial body of evidence indicates that MDSCs accumulate and exhibit abnormal immunosuppressive functions in the TME that play an important role in promoting cancer progression of cancer (Gabrilovich et al., 2012). Targeting MDSCs and reversing the immunosuppressive state of the TME are key for enhancing the effects of tumor immunotherapy. First, we discovered that the function and metabolic state of MDSCs in different tissues are heterogeneous and that the immunosuppressive function of MDSCs is enhanced after migration to the tumor site (Shen et al., 2019). In the experiment *in vitro*, we used TCCM to simulate the TME, and the results showed that TCCM upregulated its immunosuppressive effect on the proliferation of CD4^+^ T cells, promoted the release of immunosuppressive molecules, and activated the glycolytic pathway of MDSCs. Overall, increasing evidence suggests that certain factors regulate MDSCs in the TME, which should be further investigated.

Tumor cells tend to generate energy via the “Warburg effect” even under aerobic conditions and are usually accompanied by a large amount of lactate during this process. In addition to fuel energy, lactate can promote the establishment of an immunosuppressive TME (Xiong et al., 2022). Whether tumor cells can reprogram the function of MDSCs through their lactate metabolite in the TME and the specific regulatory mechanism remain unclear. In our study, we found that after treatment with lactate, both immunosuppressive function and glucose metabolism levels of MDSCs were significantly increased. Specifically, lactate enhances the immunosuppressive function of MDSCs by upregulating glycolytic activity, maintaining cell survival, and maintaining cells in an immature state. Based on these studies, it is natural to continue to advance the mechanistic research. Through whole transcriptome sequencing analysis, we found the expression of multiple genes between the lactate treatment group and control group, and these differentially expressed genes may be the key factors involved in the mechanism by which lactate regulates MDSCs function. Combined with bioinformatics analysis and verification experiments, we finally identified that expression of SGK1 greatly increased in the lactate treatment group.

In the light of that the expression of SGK1 is regulated by epigenetic factors, especially DNA methylation. Further investigation is needed to confirm the DNA methylation levels of SGK1 and elucidate the related regulatory factors. Here, we found that the methylation level of the SGK1 CpG island was significantly reduced after treatment with lactate in MDSCs using BSP and MSP. The DNA methylation level is mainly mediated by the methylated enzyme DMNTs and the demethylated enzyme TETs family (Gerecke et al., 2022), we detected the expression of DMNTs and TETs in MDSCs. The results showed that the expression of DMNTs did not change significantly; however, the expression of TET2 was significantly higher than that of TET1 and TET3, particularly after stimulation with lactate. Therefore, we hypothesized that the DNA methylation level of SGK1 may be an important mechanism for mediating the function of MDSCs via lactate regulation and that TET2 is involved in this process.

TET2 is the predominant epigenetic regulatory enzyme and an important member of the TET protein family (Lu et al., 2023; Ma et al., 2022). Previous studies have shown that TET2 regulates modifications of DNA methylation by promoting DNA demethylation by oxidizing 5-methylcytosine and the function of TET2 is closely related to embryo development and tumorigenesis. Here, we discovered that lactate could upregulate the expression of TET2 in MDSCs and that TET2 mediates the demethylation of SGK1 through a direct combination of the transcription region. Through a series of experiments, we found that the immunosuppressive function of MDSCs was significantly reduced after silencing expression of TET2 *in vitro*. Furthermore, compared with the control group, MDSCs transfected with siTET2 showed delayed tumor growth *in vivo.*

In conclusion, we revealed a novel target of SGK1 in the regulation of the immunosuppressive function of MDSCs by lactate, and clarified the important role of TET2-mediated SGK1 demethylation in anti-tumor immunity. Our findings offer unprecedented insights into the crosstalk between immune processes and metabolic regulation in MDSCs, and targeting this crosstalk is a potential novel therapeutic strategy for the treatment of tumors.

## 5 Conclusion

In the experiment, we used the subcutaneous lung cancer model, which has its limitations, including: 1. Distorted microenvironment and lack of organ specific stromal cells in the subcutaneous space; 2. The spleen becomes the main site of MDSCs amplification in subcutaneous models, however, the proportion and function of MDSCs in other key sites (such as bone marrow and tumor site) have not been evaluated, making it difficult to clarify the differences between MDSCs in different sites; 3. The systemic dynamics of MDSCs (such as migration from bone marrow to blood to spleen/tumor) need to be evaluated in the context of intact immunity. The subcutaneous transplant tumor model is difficult to reflect the systemic immune suppression associated with cancer.

Actually, both TET2 and SGK1 demonstrate potential as targeted therapies for tumors. In T-cell acute lymphoblastic leukemia (T-ALL), a phase I/II trial demonstrated that 5-aza combined with vitamin C significantly induced apoptosis in TET2-silenced cells by re-expressing TET2 and upregulating endogenous retroviruses (HERVs), thereby enhancing immunogenicity (Bensberg et al., 2021). PARP inhibitors (e.g., olaparib) exhibit efficacy in TET2-mutant acute myeloid leukemia (AML), where TET2 loss impairs homologous recombination repair (Padella et al., 2022). A phase II trial (NCT03974217) reported prolonged progression-free survival in AML patients treated with Talazoparib (clinicaltrials.gov, 2021). SGK1 inhibitors show promise in solid tumors. GSK-650394 suppresses neutrophil extracellular traps (NETs) and myeloid-derived suppressor cell infiltration, reducing colorectal cancer liver metastasis recurrence (Li et al., 2023). Natural compounds (e.g., Salvia miltiorrhiza-derived exosomes) inhibit SGK1-FOXO3a signaling, inducing autophagy/apoptosis in triple-negative breast cancer (preclinical) (Peng et al., 2025). In the future, it is necessary to rely on TET2 mutations/5hmC and SGK1 expression profiles to design precise combination schemes and accelerate the transition from mechanism research to clinical trials.

## Data Availability

The data presented in the study are deposited in the Gene Expression Omnibus repository, accession number GSE241316.

## Supplementary material

SUPPLEMENTARY FIGURE S1 T-MDSCs have higher level of glycolysis than SP-MDSCs of mouse subcutaneous lung cancer model. **(A)** The mRNA expression levels of HK2, PFK and LDHA were measured by qRT-PCR in SP-MDSCs and T-MDSCs respectively (n = 3 per group). **(B)** The mRNA expression levels of GLUT1 and HIF-1 in MDSCs were measured by qRT- PCR in SP-MDSCs and T-MDSCs respectively (n = 3 per group). **(C,D)** The glucose uptake was detected by glucose assay kit, and lactate production was examined by lactate assay kit after MDSCs were treated by TCCM for 24 h and 48 h (n = 3 per group). **(E)** The mRNA expression levels of LDHA, GLUT1 and HIF-1 in MDSCs were measured by qRT-PCR after MDSCs were treated by TCCM for 24 h and 48 h (n = 3 per group). The results are presented as means ± SEM, *p < 0.05, **p < 0.01, ***p < 0.001. T-MDSCs: MDSCs derived from the tumor tissue of mouse subcutaneous lung cancer model. SP-MDSCs: MDSCs derived from the spleens of mouse subcutaneous lung cancer model. TCCM: Tumor cell conditioned medium. HK2: hexokinase 2, PFK: phosphofructokinase, LDHA: lactate dehydrogenase A, GLUT1: glucose transporters 1, HIF-1: hypoxia inducible factor-1.

SUPPLEMENTARY FIGURE S2 Lactate enhances the level of glycolysis of MDSCs. **(A)** The mRNA expression levels of HK2, PFK and LDHA in MDSCs from wild-type mice was tested after treatment by lactate (10 μM) for 24 h (n = 3 per group). **(B)** The mRNA expression levels of GLUT1 and HIF-1 in MDSCs was tested after treatment by lactate (10 μM) for 24 h (n = 3 per group). **(C,D)** The glucose consumption was detected by glucose assay kit, and lactate production was examined by lactate assay kit after MDSCs were treated by lactate (10 μM) for 24 h (n = 3 per group). The results are presented as means ± SEM, *p < 0.05, **p < 0.01. LA: lactate, HK2: hexokinase 2, PFK: phosphofructokinase, GLUT1: glucose transporters 1, HIF-1: hypoxia inducible factor-1.

SUPPLEMENTARY FIGURE S3 After the glycogenic pathway is suppressed, the expression of inhibitory molecules in MDSCs were declined. **(A)** The mRNA expression levels of HK, PFK and LDHA in MDSCs from wild-type mice was tested by qRT-PCR after treatment of 2-DG (10 μM) (n = 3 per group). **(B)** The mRNA expression levels of GLUT1 and HIF-1 in MDSCs was tested by qRT-PCR after treatment of 2-DG (10 μM) (n = 3 per group). **(C,D)** The glucose consumption was detected by glucose assay kit, and lactate production was examined by lactate assay kit (n = 3 per group). **(E,F)** The activity of Arg-1 was measured by a QuantiChrom arginase assay kit and the concentration of NO was determined by a Griess reagent system kit (n = 3 per group). The results are presented as means ± SEM, *p < 0.05, **p < 0.01, ***p < 0.001. LA: MDSCs were incubated with lactate, LA+2-DG: MDSCs were treated with lactate and 2-DG, 2-DG: 2-deoxy-D-glucose.
