# Supplementary figures and images for: Lactate modulates the function of myeloid-derived suppressor cells via Ten-Eleven-Translocation-2-mediated demethylation of glucocorticoid-inducible kinase 1 in lung cancer model
Source: Front Cell Dev Biol. 2025 Aug 22;13:1565993. doi: 10.3389/fcell.2025.1565993 (PMC12411531; doi:10.3389/fcell.2025.1565993)

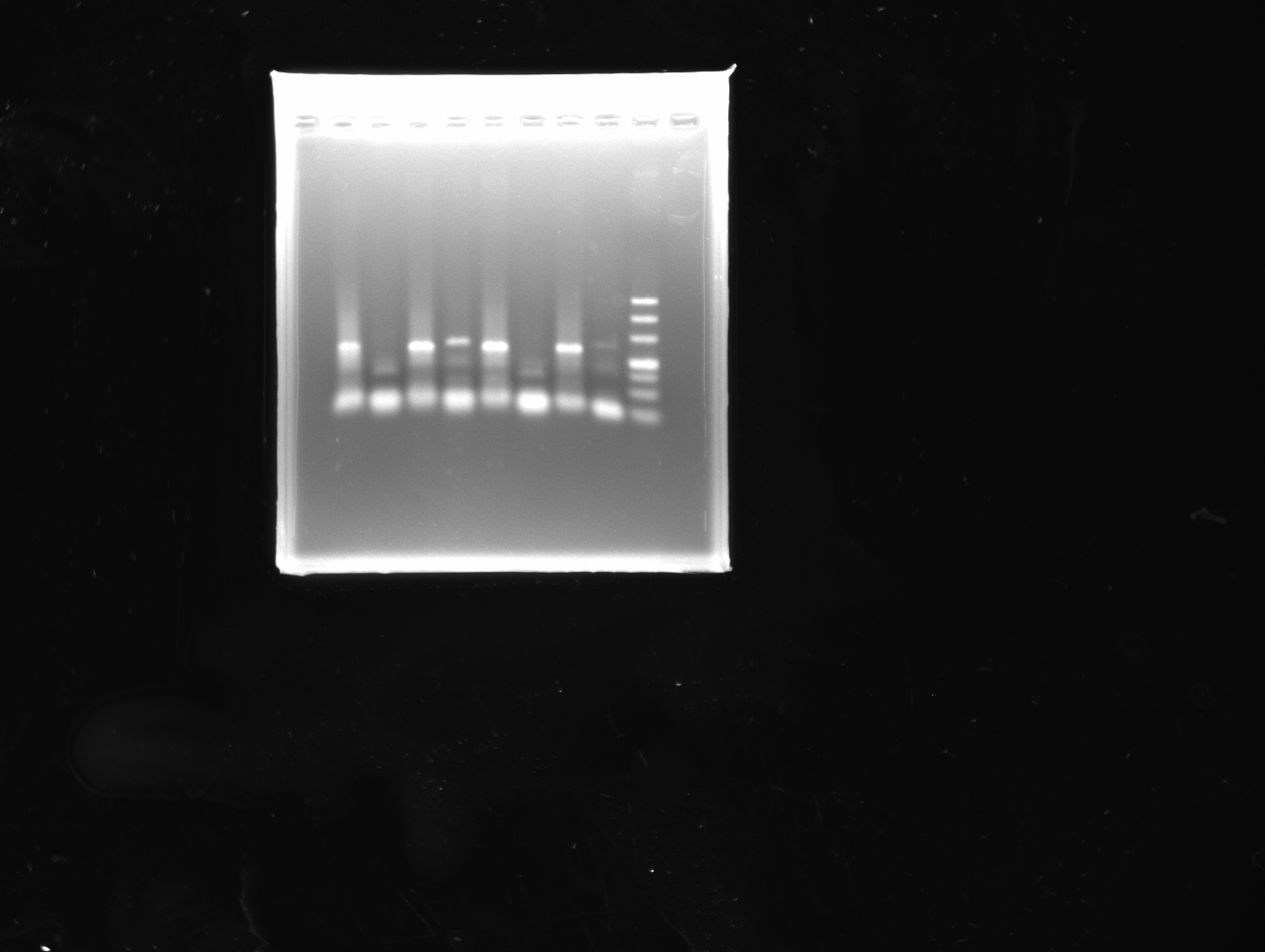

Supplement: Supplementary file 1 [file DataSheet1.zip › Original Data/Figure 4/F4-E.tif]
